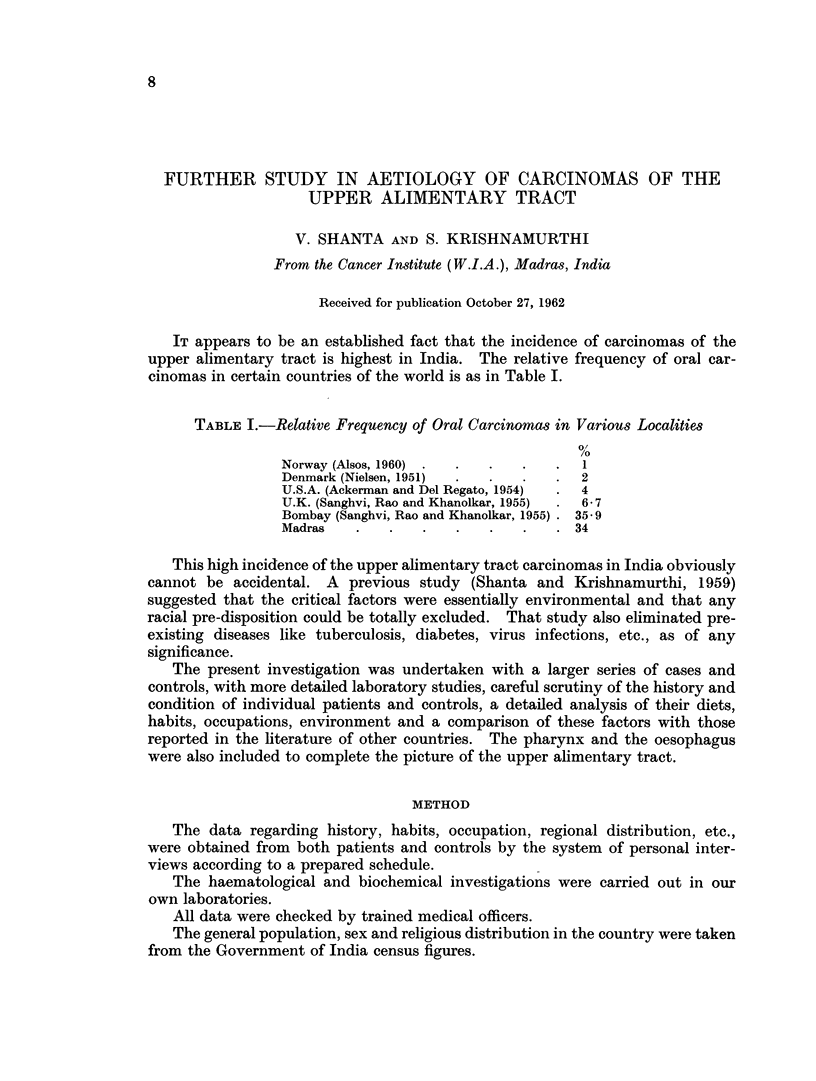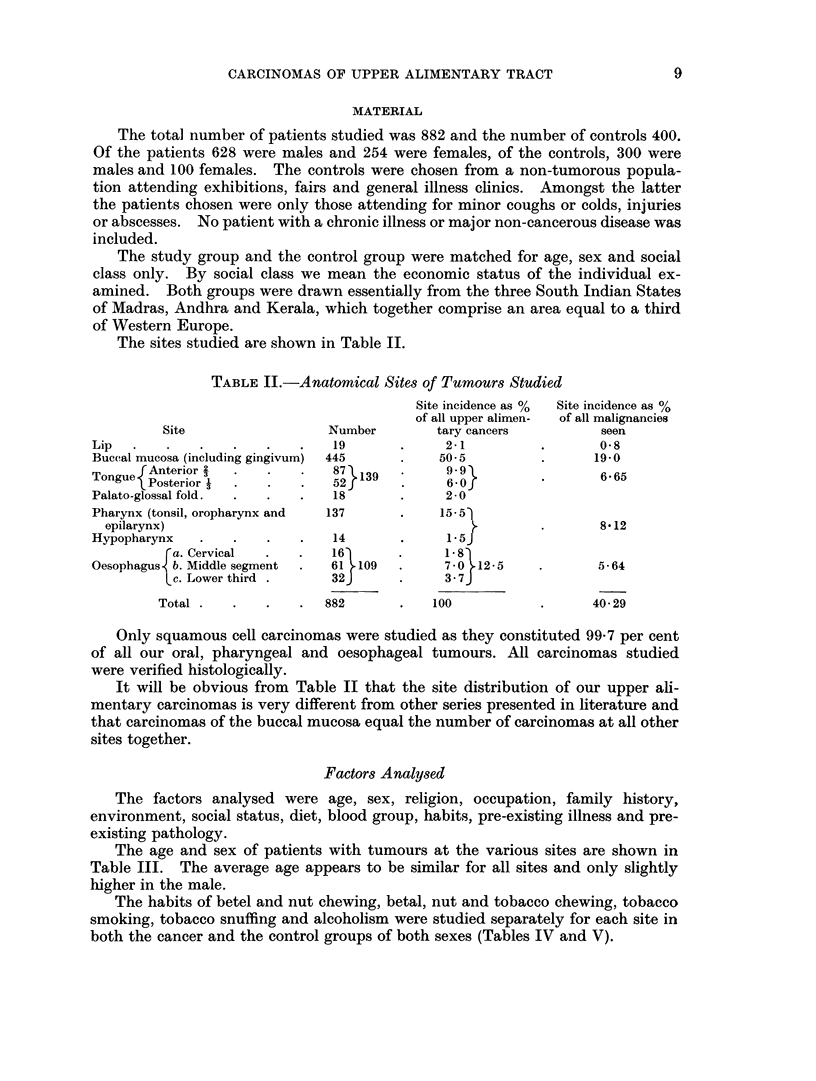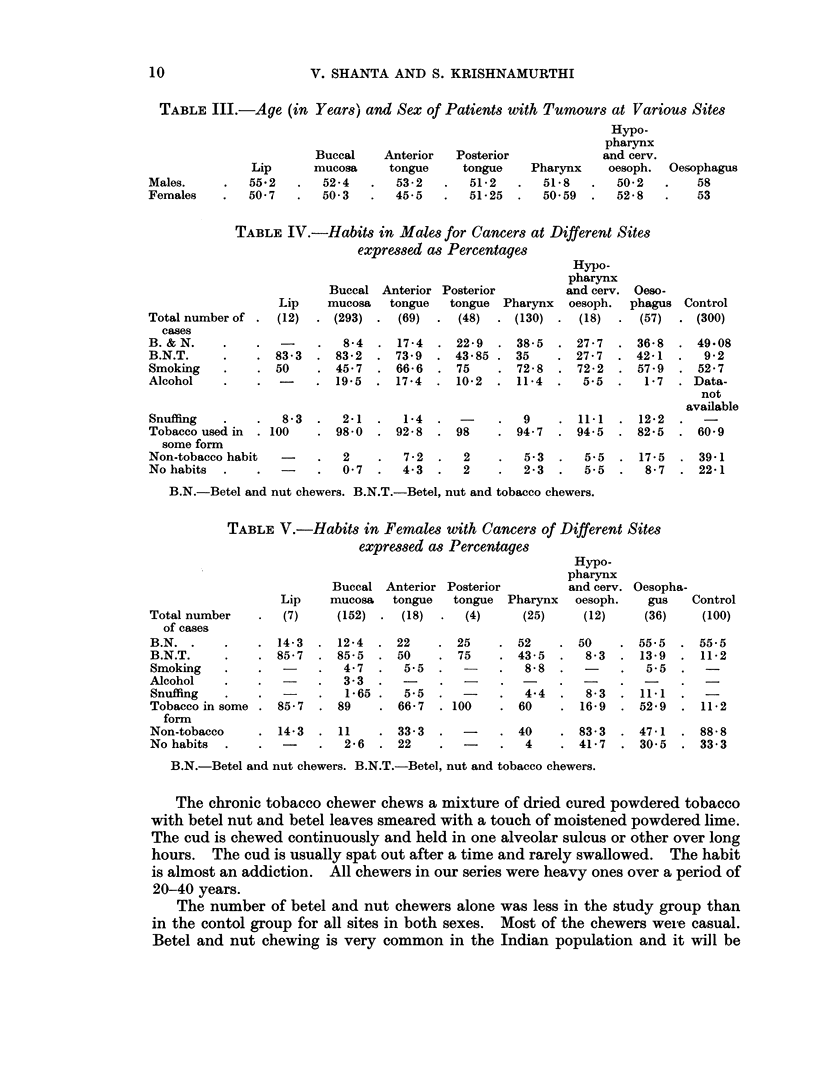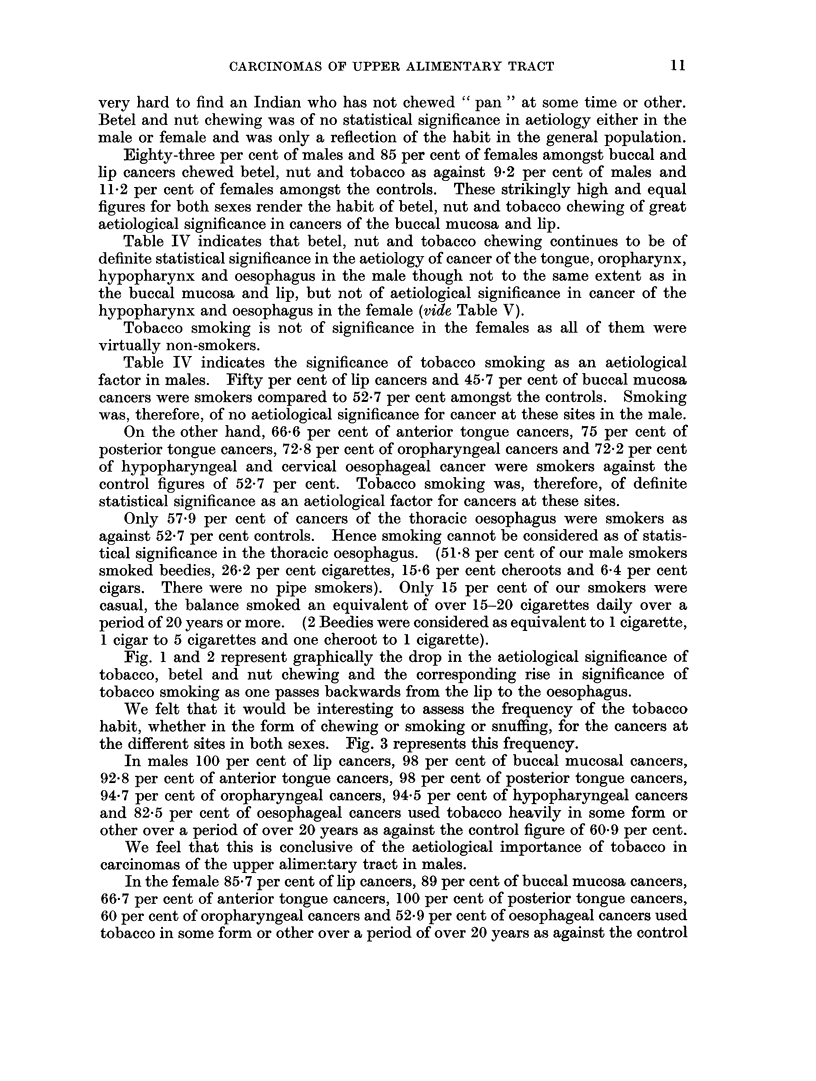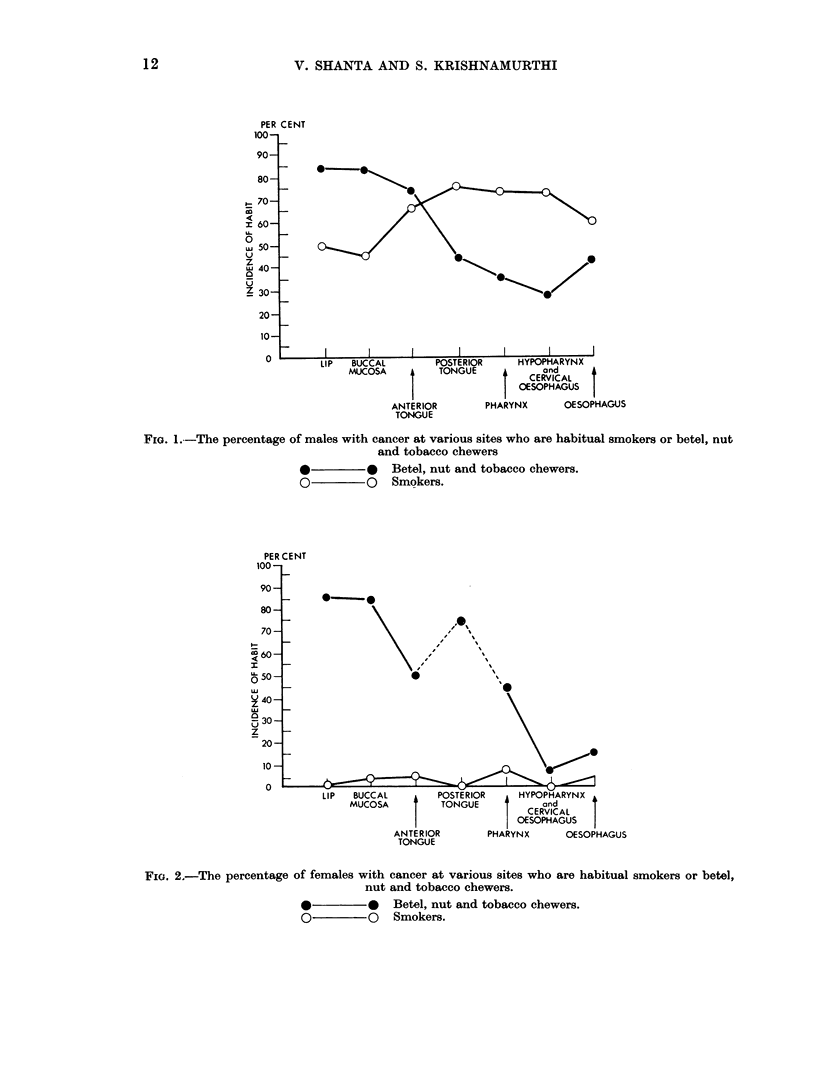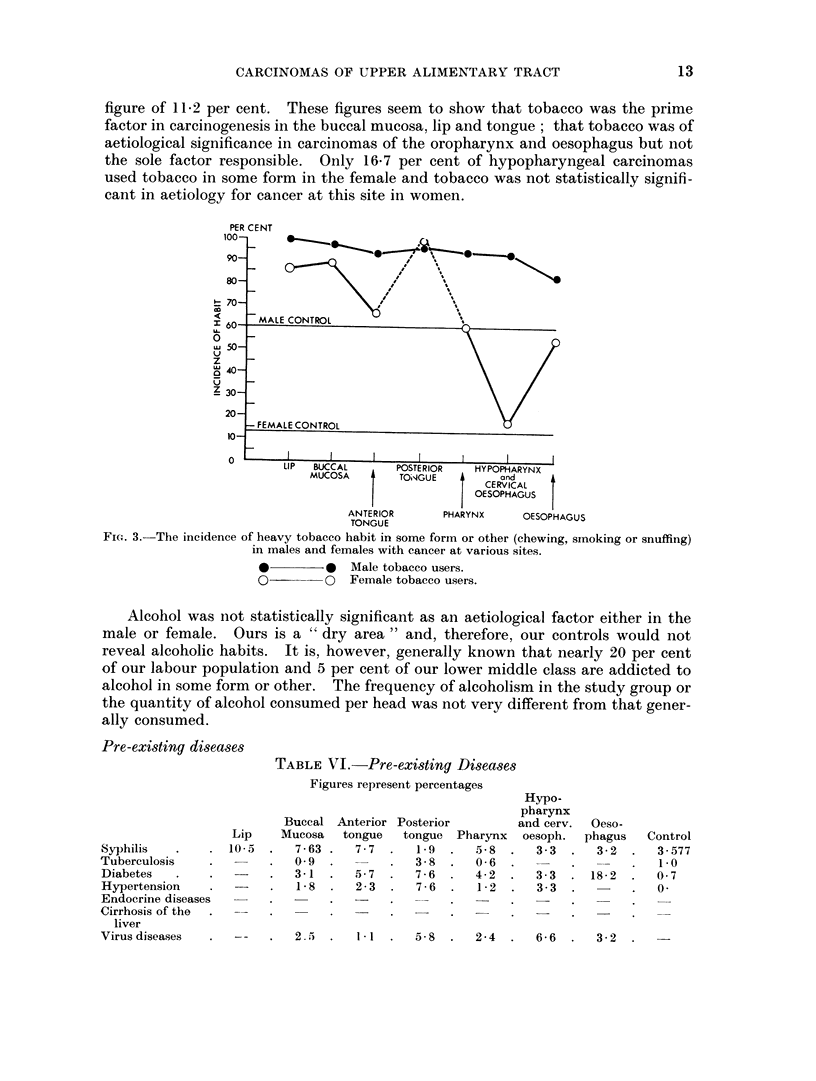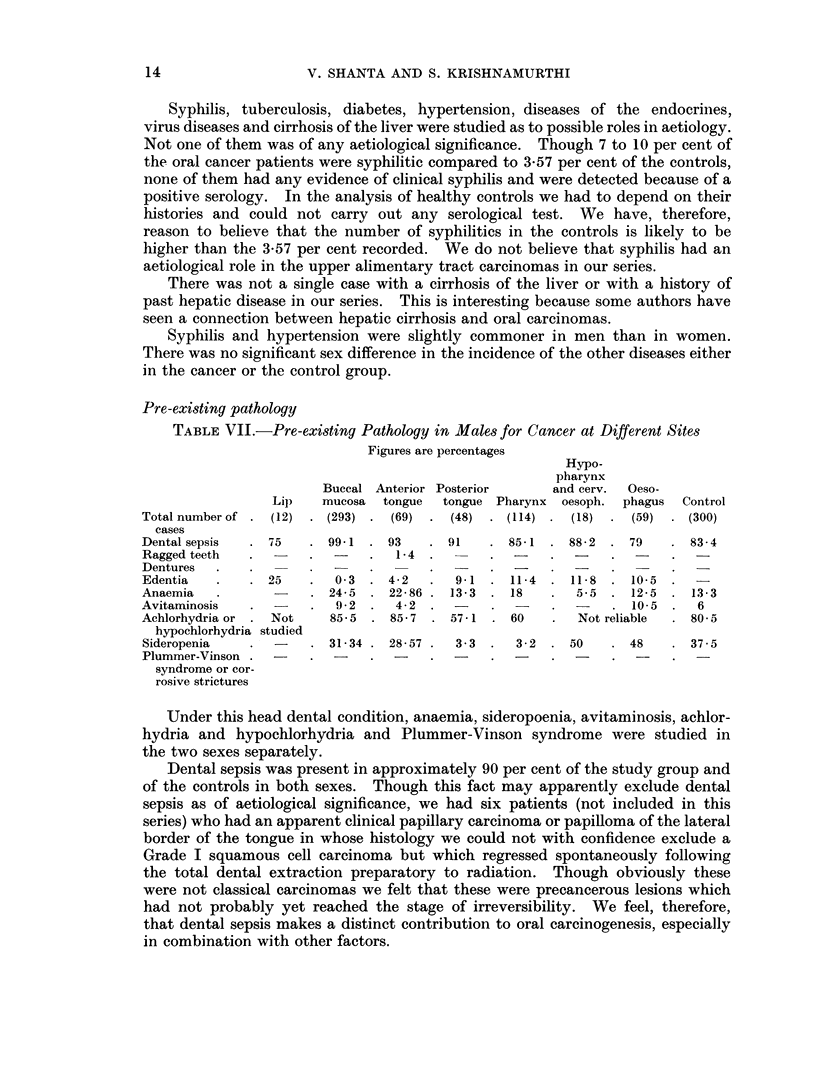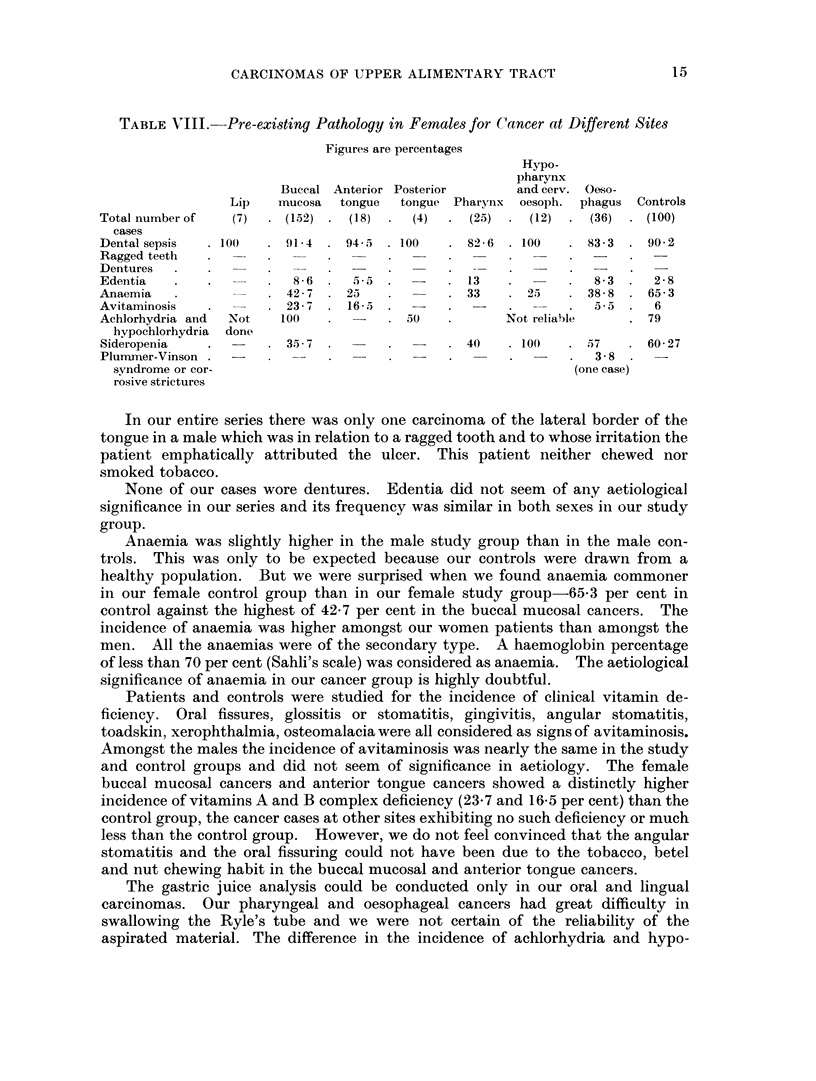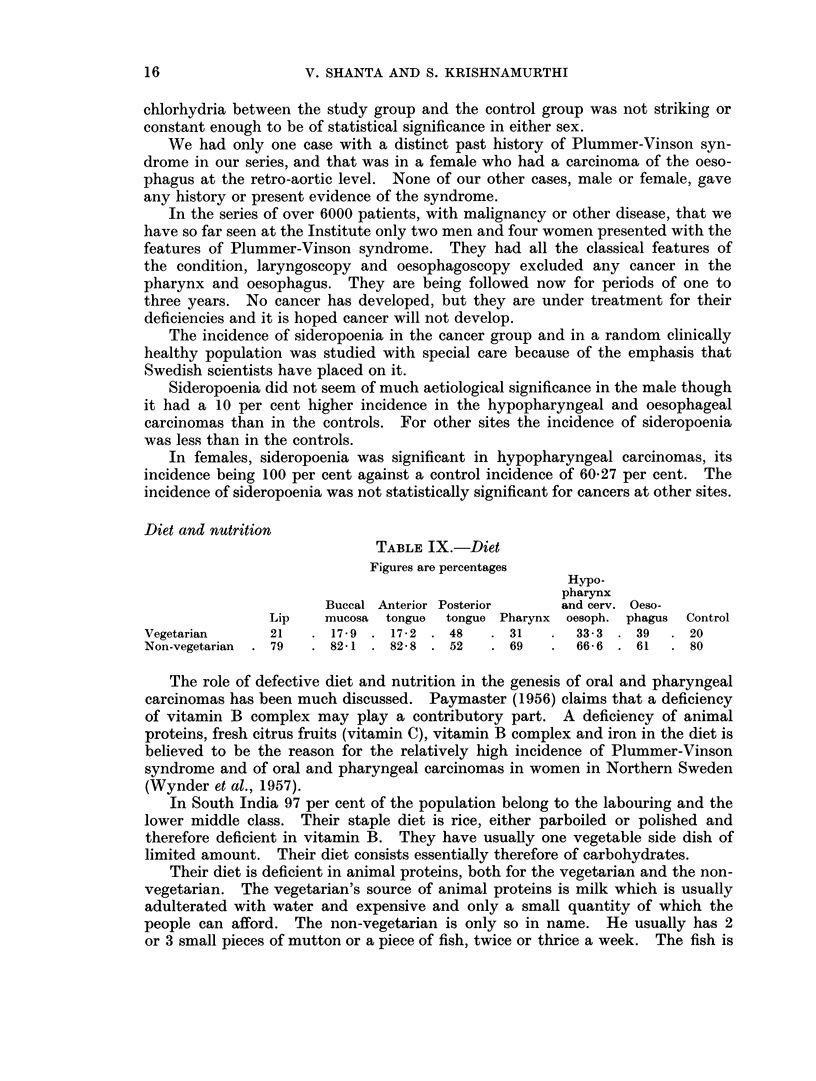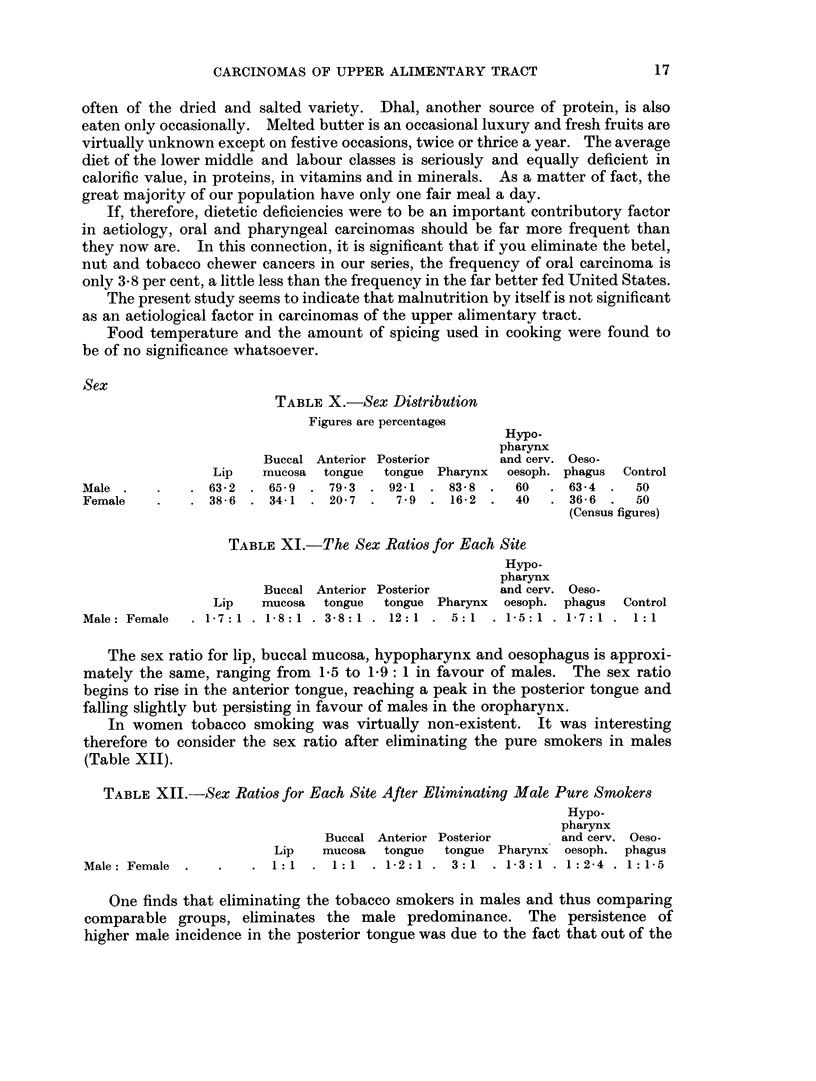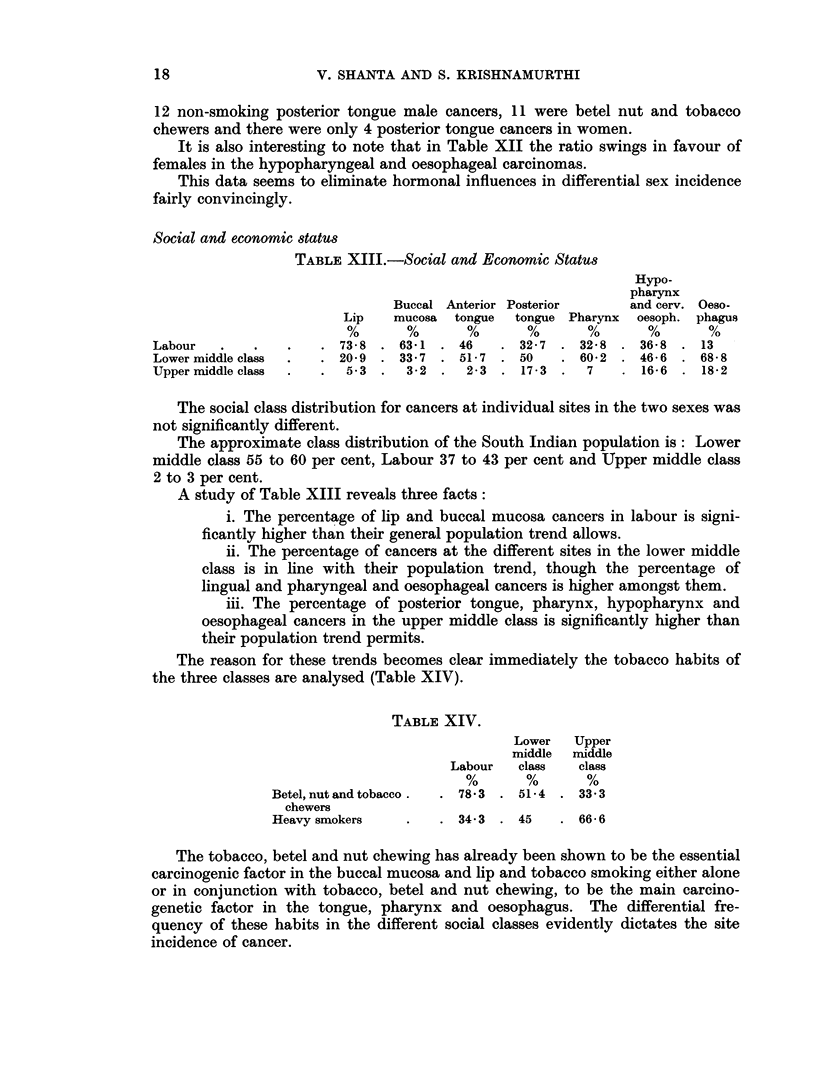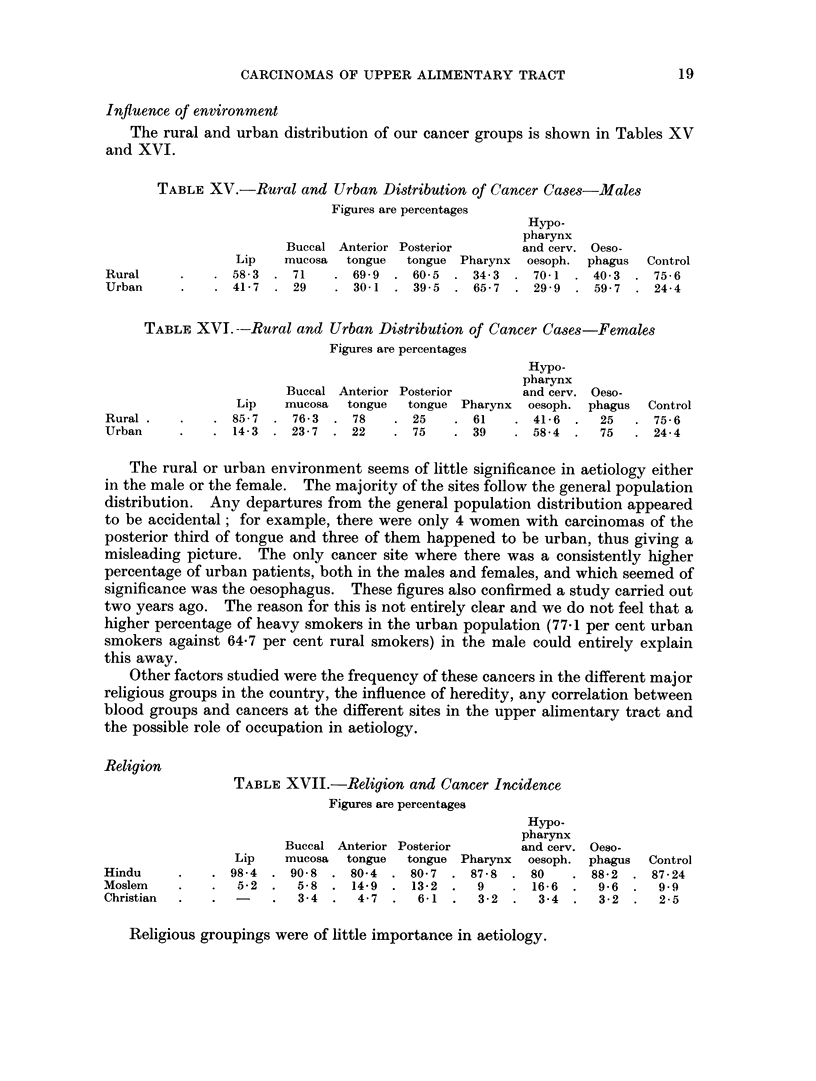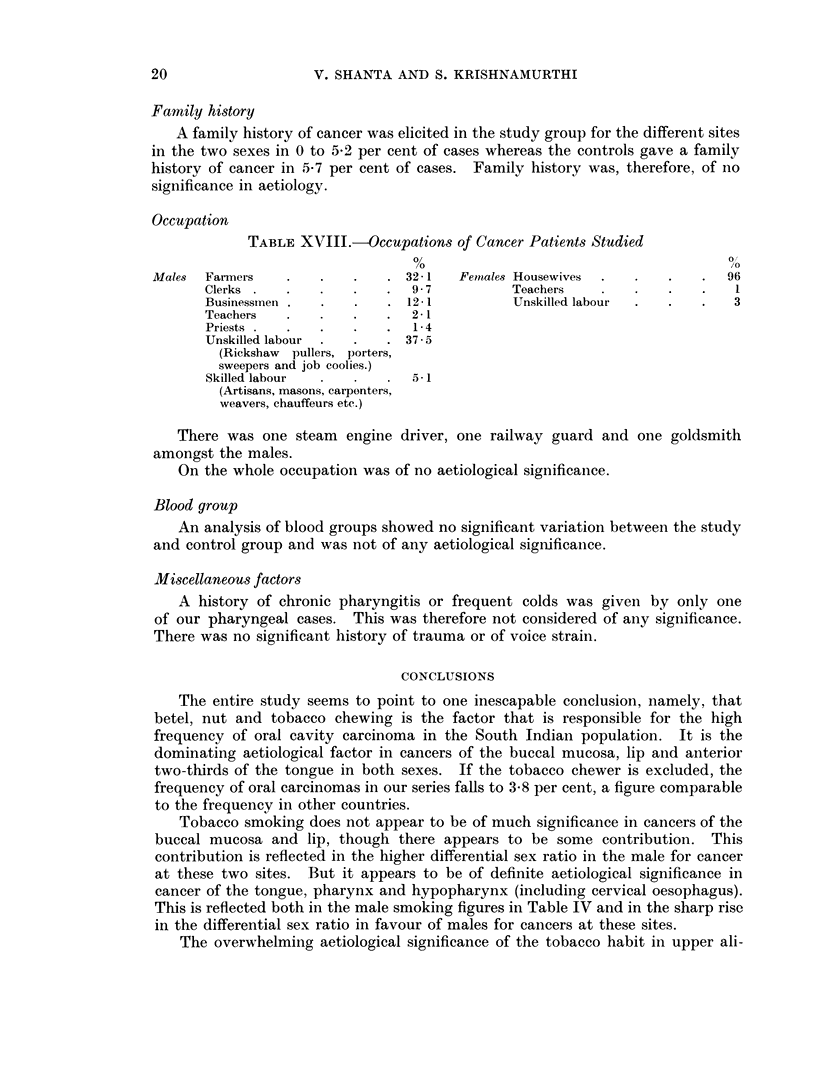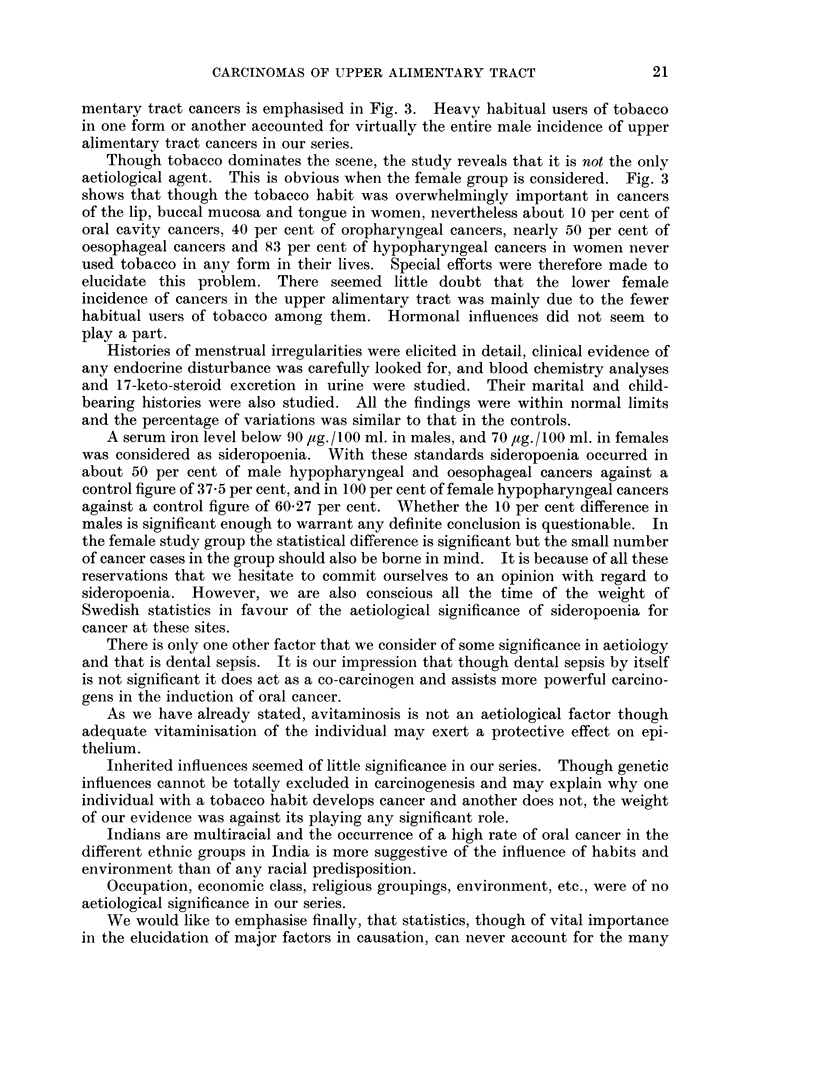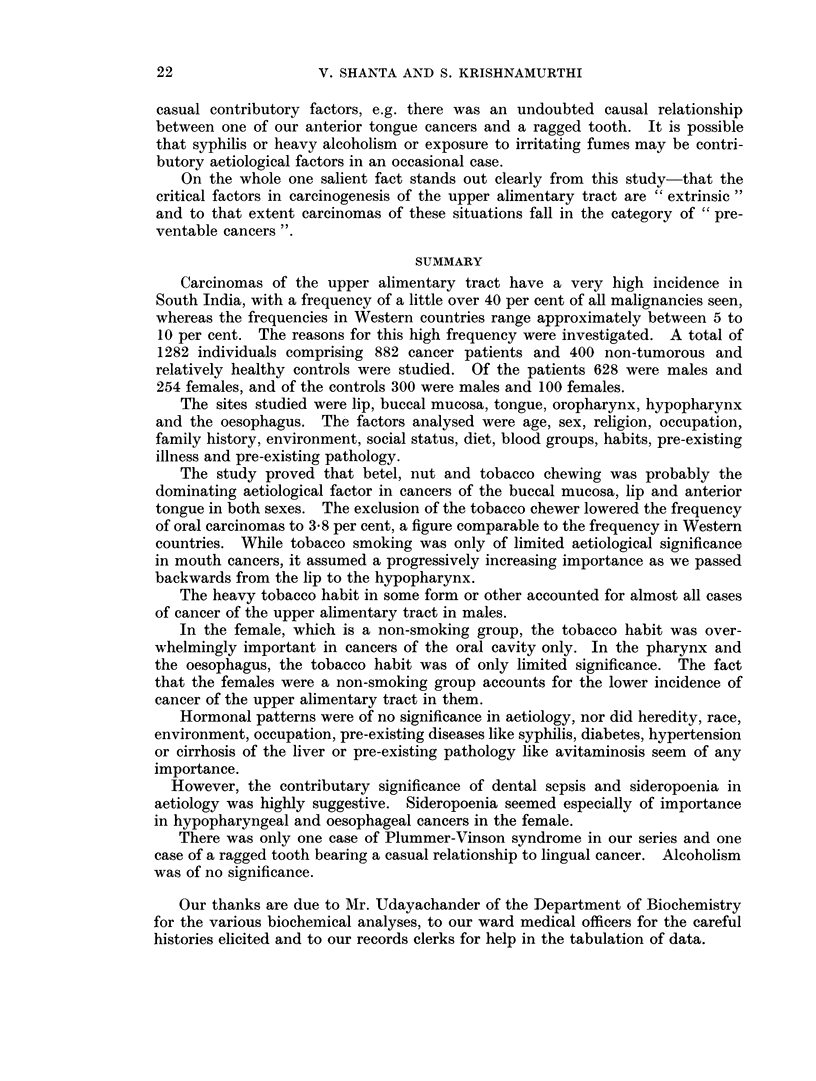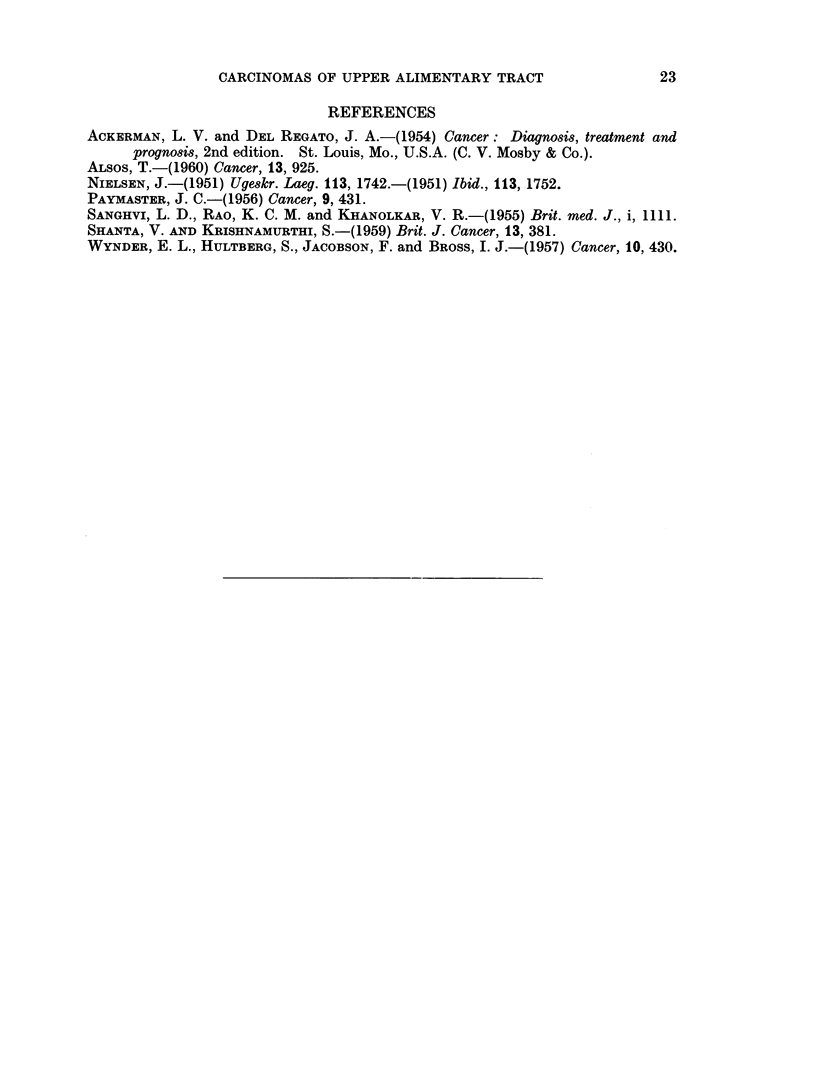# Further Study in Aetiology of Carcinomas of the Upper Alimentary Tract

**DOI:** 10.1038/bjc.1963.2

**Published:** 1963-03

**Authors:** V. Shanta, S. Krishnamurthi


### FURTHER STUDY IN AETIOLOGY OF CARCINOMAS OF THE UPPER ALIMENTARY TRACT

V. SHANTA AND S. KRISHNAMURTHI

*From the Cancer Institute (W.I.A.), Madras, India*

Received for publication October 27, 1962

IT appears to be an established fact that the incidence of carcinomas of the upper alimentary tract is highest in India. The relative frequency of oral carcinomas in certain countries of the world is as in Table I.

TABLE I.—*Relative Frequency of Oral Carcinomas in Various Localities*

| | % |
|---|---|
| Norway (Alsos, 1960) | 1 |
| Denmark (Nielsen, 1951) | 2 |
| U.S.A. (Ackerman and Del Regato, 1954) | 4 |
| U.K. (Sanghvi, Rao and Khanolkar, 1955) | 6·7 |
| Bombay (Sanghvi, Rao and Khanolkar, 1955) | 35·9 |
| Madras | 34 |

This high incidence of the upper alimentary tract carcinomas in India obviously cannot be accidental. A previous study (Shanta and Krishnamurthi, 1959) suggested that the critical factors were essentially environmental and that any racial pre-disposition could be totally excluded. That study also eliminated pre-existing diseases like tuberculosis, diabetes, virus infections, etc., as of any significance.

The present investigation was undertaken with a larger series of cases and controls, with more detailed laboratory studies, careful scrutiny of the history and condition of individual patients and controls, a detailed analysis of their diets, habits, occupations, environment and a comparison of these factors with those reported in the literature of other countries. The pharynx and the oesophagus were also included to complete the picture of the upper alimentary tract.

### METHOD

The data regarding history, habits, occupation, regional distribution, etc., were obtained from both patients and controls by the system of personal interviews according to a prepared schedule.

The haematological and biochemical investigations were carried out in our own laboratories.

All data were checked by trained medical officers.

The general population, sex and religious distribution in the country were taken from the Government of India census figures.

### MATERIAL

The total number of patients studied was 882 and the number of controls 400. Of the patients 628 were males and 254 were females, of the controls, 300 were males and 100 females. The controls were chosen from a non-tumorous population attending exhibitions, fairs and general illness clinics. Amongst the latter the patients chosen were only those attending for minor coughs or colds, injuries or abscesses. No patient with a chronic illness or major non-cancerous disease was included.

The study group and the control group were matched for age, sex and social class only. By social class we mean the economic status of the individual examined. Both groups were drawn essentially from the three South Indian States of Madras, Andhra and Kerala, which together comprise an area equal to a third of Western Europe.

The sites studied are shown in Table II.

TABLE II.—*Anatomical Sites of Tumours Studied*

| Site | | Number | | Site incidence as % of all upper alimentary cancers | | Site incidence as % of all malignancies seen |
|---|---|---|---|---|---|---|
| Lip | 19 | | 2·1 | | 0·8 |
| Buccal mucosa (including gingivum) | 445 | | 50·5 | | 19·0 |
| Tongue: Anterior $\frac{2}{3}$ | 87 | 139 | 9·9 | | 6·65 |
| Tongue: Posterior $\frac{1}{3}$ | 52 | | 6·0 | | |
| Palato-glossal fold | 18 | | 2·0 | | |
| Pharynx (tonsil, oropharynx and epilarynx) | 137 | | 15·5 | | 8·12 |
| Hypopharynx | 14 | | 1·5 | | |
| Oesophagus: a. Cervical | 16 | 109 | 1·8 | 12·5 | 5·64 |
| Oesophagus: b. Middle segment | 61 | | 7·0 | | |
| Oesophagus: c. Lower third | 32 | | 3·7 | | |
| Total | 882 | | 100 | | 40·29 |

Only squamous cell carcinomas were studied as they constituted 99·7 per cent of all our oral, pharyngeal and oesophageal tumours. All carcinomas studied were verified histologically.

It will be obvious from Table II that the site distribution of our upper alimentary carcinomas is very different from other series presented in literature and that carcinomas of the buccal mucosa equal the number of carcinomas at all other sites together.

### *Factors Analysed*

The factors analysed were age, sex, religion, occupation, family history, environment, social status, diet, blood group, habits, pre-existing illness and pre-existing pathology.

The age and sex of patients with tumours at the various sites are shown in Table III. The average age appears to be similar for all sites and only slightly higher in the male.

The habits of betel and nut chewing, betal, nut and tobacco chewing, tobacco smoking, tobacco snuffing and alcoholism were studied separately for each site in both the cancer and the control groups of both sexes (Tables IV and V).

TABLE III.—*Age (in Years) and Sex of Patients with Tumours at Various Sites*

| | Lip | Buccal mucosa | Anterior tongue | Posterior tongue | Pharynx | Hypo-pharynx and cerv. oesoph. | Oesophagus |
|---|---|---|---|---|---|---|---|
| Males | 55·2 | 52·4 | 53·2 | 51·2 | 51·8 | 50·2 | 58 |
| Females | 50·7 | 50·3 | 45·5 | 51·25 | 50·59 | 52·8 | 53 |

TABLE IV.—*Habits in Males for Cancers at Different Sites expressed as Percentages*

| | Lip | Buccal mucosa | Anterior tongue | Posterior tongue | Pharynx | Hypo-pharynx and cerv. oesoph. | Oeso-phagus | Control |
|---|---|---|---|---|---|---|---|---|
| Total number of cases | (12) | (293) | (69) | (48) | (130) | (18) | (57) | (300) |
| B. & N. | — | 8·4 | 17·4 | 22·9 | 38·5 | 27·7 | 36·8 | 49·08 |
| B.N.T. | 83·3 | 83·2 | 73·9 | 43·85 | 35 | 27·7 | 42·1 | 9·2 |
| Smoking | 50 | 45·7 | 66·6 | 75 | 72·8 | 72·2 | 57·9 | 52·7 |
| Alcohol | — | 19·5 | 17·4 | 10·2 | 11·4 | 5·5 | 1·7 | Data not available |
| Snuffing | 8·3 | 2·1 | 1·4 | — | 9 | 11·1 | 12·2 | — |
| Tobacco used in some form | 100 | 98·0 | 92·8 | 98 | 94·7 | 94·5 | 82·5 | 60·9 |
| Non-tobacco habit | — | 2 | 7·2 | 2 | 5·3 | 5·5 | 17·5 | 39·1 |
| No habits | — | 0·7 | 4·3 | 2 | 2·3 | 5·5 | 8·7 | 22·1 |

B.N.—Betel and nut chewers. B.N.T.—Betel, nut and tobacco chewers.

TABLE V.—*Habits in Females with Cancers of Different Sites expressed as Percentages*

| | Lip | Buccal mucosa | Anterior tongue | Posterior tongue | Pharynx | Hypo-pharynx and cerv. oesoph. | Oesopha-gus | Control |
|---|---|---|---|---|---|---|---|---|
| Total number of cases | (7) | (152) | (18) | (4) | (25) | (12) | (36) | (100) |
| B.N. | 14·3 | 12·4 | 22 | 25 | 52 | 50 | 55·5 | 55·5 |
| B.N.T. | 85·7 | 85·5 | 50 | 75 | 43·5 | 8·3 | 13·9 | 11·2 |
| Smoking | — | 4·7 | 5·5 | — | 8·8 | — | 5·5 | — |
| Alcohol | — | 3·3 | — | — | — | — | — | — |
| Snuffing | — | 1·65 | 5·5 | — | 4·4 | 8·3 | 11·1 | — |
| Tobacco in some form | 85·7 | 89 | 66·7 | 100 | 60 | 16·9 | 52·9 | 11·2 |
| Non-tobacco | 14·3 | 11 | 33·3 | — | 40 | 83·3 | 47·1 | 88·8 |
| No habits | — | 2·6 | 22 | — | 4 | 41·7 | 30·5 | 33·3 |

B.N.—Betel and nut chewers. B.N.T.—Betel, nut and tobacco chewers.

The chronic tobacco chewer chews a mixture of dried cured powdered tobacco with betel nut and betel leaves smeared with a touch of moistened powdered lime. The cud is chewed continuously and held in one alveolar sulcus or other over long hours. The cud is usually spat out after a time and rarely swallowed. The habit is almost an addiction. All chewers in our series were heavy ones over a period of 20–40 years.

The number of betel and nut chewers alone was less in the study group than in the contol group for all sites in both sexes. Most of the chewers were casual. Betel and nut chewing is very common in the Indian population and it will be very hard to find an Indian who has not chewed " pan " at some time or other. Betel and nut chewing was of no statistical significance in aetiology either in the male or female and was only a reflection of the habit in the general population.

Eighty-three per cent of males and 85 per cent of females amongst buccal and lip cancers chewed betel, nut and tobacco as against 9·2 per cent of males and 11·2 per cent of females amongst the controls. These strikingly high and equal figures for both sexes render the habit of betel, nut and tobacco chewing of great aetiological significance in cancers of the buccal mucosa and lip.

Table IV indicates that betel, nut and tobacco chewing continues to be of definite statistical significance in the aetiology of cancer of the tongue, oropharynx, hypopharynx and oesophagus in the male though not to the same extent as in the buccal mucosa and lip, but not of aetiological significance in cancer of the hypopharynx and oesophagus in the female (*vide* Table V).

Tobacco smoking is not of significance in the females as all of them were virtually non-smokers.

Table IV indicates the significance of tobacco smoking as an aetiological factor in males. Fifty per cent of lip cancers and 45·7 per cent of buccal mucosa cancers were smokers compared to 52·7 per cent amongst the controls. Smoking was, therefore, of no aetiological significance for cancer at these sites in the male.

On the other hand, 66·6 per cent of anterior tongue cancers, 75 per cent of posterior tongue cancers, 72·8 per cent of oropharyngeal cancers and 72·2 per cent of hypopharyngeal and cervical oesophageal cancer were smokers against the control figures of 52·7 per cent. Tobacco smoking was, therefore, of definite statistical significance as an aetiological factor for cancers at these sites.

Only 57·9 per cent of cancers of the thoracic oesophagus were smokers as against 52·7 per cent controls. Hence smoking cannot be considered as of statistical significance in the thoracic oesophagus. (51·8 per cent of our male smokers smoked beedies, 26·2 per cent cigarettes, 15·6 per cent cheroots and 6·4 per cent cigars. There were no pipe smokers). Only 15 per cent of our smokers were casual, the balance smoked an equivalent of over 15–20 cigarettes daily over a period of 20 years or more. (2 Beedies were considered as equivalent to 1 cigarette, 1 cigar to 5 cigarettes and one cheroot to 1 cigarette).

Fig. 1 and 2 represent graphically the drop in the aetiological significance of tobacco, betel and nut chewing and the corresponding rise in significance of tobacco smoking as one passes backwards from the lip to the oesophagus.

We felt that it would be interesting to assess the frequency of the tobacco habit, whether in the form of chewing or smoking or snuffing, for the cancers at the different sites in both sexes. Fig. 3 represents this frequency.

In males 100 per cent of lip cancers, 98 per cent of buccal mucosal cancers, 92·8 per cent of anterior tongue cancers, 98 per cent of posterior tongue cancers, 94·7 per cent of oropharyngeal cancers, 94·5 per cent of hypopharyngeal cancers and 82·5 per cent of oesophageal cancers used tobacco heavily in some form or other over a period of over 20 years as against the control figure of 60·9 per cent.

We feel that this is conclusive of the aetiological importance of tobacco in carcinomas of the upper alimentary tract in males.

In the female 85·7 per cent of lip cancers, 89 per cent of buccal mucosa cancers, 66·7 per cent of anterior tongue cancers, 100 per cent of posterior tongue cancers, 60 per cent of oropharyngeal cancers and 52·9 per cent of oesophageal cancers used tobacco in some form or other over a period of over 20 years as against the control

Fig. 1.—The percentage of males with cancer at various sites who are habitual smokers or betel, nut and tobacco chewers

●———● Betel, nut and tobacco chewers.

○———○ Smokers.

Fig. 2.—The percentage of females with cancer at various sites who are habitual smokers or betel, nut and tobacco chewers.

●———● Betel, nut and tobacco chewers.

○———○ Smokers.

figure of 11·2 per cent. These figures seem to show that tobacco was the prime factor in carcinogenesis in the buccal mucosa, lip and tongue ; that tobacco was of aetiological significance in carcinomas of the oropharynx and oesophagus but not the sole factor responsible. Only 16·7 per cent of hypopharyngeal carcinomas used tobacco in some form in the female and tobacco was not statistically significant in aetiology for cancer at this site in women.

FIG. 3.—The incidence of heavy tobacco habit in some form or other (chewing, smoking or snuffing) in males and females with cancer at various sites.

●———● Male tobacco users.

○———○ Female tobacco users.

Alcohol was not statistically significant as an aetiological factor either in the male or female. Ours is a " dry area " and, therefore, our controls would not reveal alcoholic habits. It is, however, generally known that nearly 20 per cent of our labour population and 5 per cent of our lower middle class are addicted to alcohol in some form or other. The frequency of alcoholism in the study group or the quantity of alcohol consumed per head was not very different from that generally consumed.

*Pre-existing diseases*

TABLE VI.—*Pre-existing Diseases*

Figures represent percentages

| | Lip | Buccal Mucosa | Anterior tongue | Posterior tongue | Pharynx | Hypo-pharynx and cerv. oesoph. | Oeso-phagus | Control |
|---|---|---|---|---|---|---|---|---|
| Syphilis | 10·5 | 7·63 | 7·7 | 1·9 | 5·8 | 3·3 | 3·2 | 3·577 |
| Tuberculosis | — | 0·9 | — | 3·8 | 0·6 | — | — | 1·0 |
| Diabetes | — | 3·1 | 5·7 | 7·6 | 4·2 | 3·3 | 18·2 | 0·7 |
| Hypertension | — | 1·8 | 2·3 | 7·6 | 1·2 | 3·3 | — | 0· |
| Endocrine diseases | — | — | — | — | — | — | — | — |
| Cirrhosis of the liver | — | — | — | — | — | — | — | — |
| Virus diseases | — | 2.5 | 1·1 | 5·8 | 2·4 | 6·6 | 3·2 | — |

Syphilis, tuberculosis, diabetes, hypertension, diseases of the endocrines, virus diseases and cirrhosis of the liver were studied as to possible roles in aetiology. Not one of them was of any aetiological significance. Though 7 to 10 per cent of the oral cancer patients were syphilitic compared to 3·57 per cent of the controls, none of them had any evidence of clinical syphilis and were detected because of a positive serology. In the analysis of healthy controls we had to depend on their histories and could not carry out any serological test. We have, therefore, reason to believe that the number of syphilitics in the controls is likely to be higher than the 3·57 per cent recorded. We do not believe that syphilis had an aetiological role in the upper alimentary tract carcinomas in our series.

There was not a single case with a cirrhosis of the liver or with a history of past hepatic disease in our series. This is interesting because some authors have seen a connection between hepatic cirrhosis and oral carcinomas.

Syphilis and hypertension were slightly commoner in men than in women. There was no significant sex difference in the incidence of the other diseases either in the cancer or the control group.

*Pre-existing pathology*

TABLE VII.—*Pre-existing Pathology in Males for Cancer at Different Sites*

Figures are percentages

| | Lip | Buccal mucosa | Anterior tongue | Posterior tongue | Pharynx | Hypo-pharynx and cerv. oesoph. | Oeso-phagus | Control |
|---|---|---|---|---|---|---|---|---|
| Total number of cases | (12) | (293) | (69) | (48) | (114) | (18) | (59) | (300) |
| Dental sepsis | 75 | 99·1 | 93 | 91 | 85·1 | 88·2 | 79 | 83·4 |
| Ragged teeth | — | — | 1·4 | — | — | — | — | — |
| Dentures | — | — | — | — | — | — | — | — |
| Edentia | 25 | 0·3 | 4·2 | 9·1 | 11·4 | 11·8 | 10·5 | — |
| Anaemia | — | 24·5 | 22·86 | 13·3 | 18 | 5·5 | 12·5 | 13·3 |
| Avitaminosis | — | 9·2 | 4·2 | — | — | — | 10·5 | 6 |
| Achlorhydria or hypochlorhydria | Not studied | 85·5 | 85·7 | 57·1 | 60 | Not reliable | | 80·5 |
| Sideropenia | — | 31·34 | 28·57 | 3·3 | 3·2 | 50 | 48 | 37·5 |
| Plummer-Vinson syndrome or corrosive strictures | — | — | — | — | — | — | — | — |

Under this head dental condition, anaemia, sideropoenia, avitaminosis, achlorhydria and hypochlorhydria and Plummer-Vinson syndrome were studied in the two sexes separately.

Dental sepsis was present in approximately 90 per cent of the study group and of the controls in both sexes. Though this fact may apparently exclude dental sepsis as of aetiological significance, we had six patients (not included in this series) who had an apparent clinical papillary carcinoma or papilloma of the lateral border of the tongue in whose histology we could not with confidence exclude a Grade I squamous cell carcinoma but which regressed spontaneously following the total dental extraction preparatory to radiation. Though obviously these were not classical carcinomas we felt that these were precancerous lesions which had not probably yet reached the stage of irreversibility. We feel, therefore, that dental sepsis makes a distinct contribution to oral carcinogenesis, especially in combination with other factors.

TABLE VIII.—*Pre-existing Pathology in Females for Cancer at Different Sites*

Figures are percentages

| | Lip | Buccal mucosa | Anterior tongue | Posterior tongue | Pharynx | Hypo-pharynx and cerv. oesoph. | Oeso-phagus | Controls |
|---|---|---|---|---|---|---|---|---|
| Total number of cases | (7) | (152) | (18) | (4) | (25) | (12) | (36) | (100) |
| Dental sepsis | 100 | 91·4 | 94·5 | 100 | 82·6 | 100 | 83·3 | 90·2 |
| Ragged teeth | — | — | — | — | — | — | — | — |
| Dentures | — | — | — | — | — | — | — | — |
| Edentia | — | 8·6 | 5·5 | — | 13 | — | 8·3 | 2·8 |
| Anaemia | — | 42·7 | 25 | — | 33 | 25 | 38·8 | 65·3 |
| Avitaminosis | — | 23·7 | 16·5 | — | — | — | 5·5 | 6 |
| Achlorhydria and hypochlorhydria | Not done | 100 | — | 50 | Not reliable | | | 79 |
| Sideropenia | — | 35·7 | — | — | 40 | 100 | 57 | 60·27 |
| Plummer-Vinson syndrome or corrosive strictures | — | — | — | — | — | — | 3·8 (one case) | — |

In our entire series there was only one carcinoma of the lateral border of the tongue in a male which was in relation to a ragged tooth and to whose irritation the patient emphatically attributed the ulcer. This patient neither chewed nor smoked tobacco.

None of our cases wore dentures. Edentia did not seem of any aetiological significance in our series and its frequency was similar in both sexes in our study group.

Anaemia was slightly higher in the male study group than in the male controls. This was only to be expected because our controls were drawn from a healthy population. But we were surprised when we found anaemia commoner in our female control group than in our female study group—65·3 per cent in control against the highest of 42·7 per cent in the buccal mucosal cancers. The incidence of anaemia was higher amongst our women patients than amongst the men. All the anaemias were of the secondary type. A haemoglobin percentage of less than 70 per cent (Sahli's scale) was considered as anaemia. The aetiological significance of anaemia in our cancer group is highly doubtful.

Patients and controls were studied for the incidence of clinical vitamin deficiency. Oral fissures, glossitis or stomatitis, gingivitis, angular stomatitis, toadskin, xerophthalmia, osteomalacia were all considered as signs of avitaminosis. Amongst the males the incidence of avitaminosis was nearly the same in the study and control groups and did not seem of significance in aetiology. The female buccal mucosal cancers and anterior tongue cancers showed a distinctly higher incidence of vitamins A and B complex deficiency (23·7 and 16·5 per cent) than the control group, the cancer cases at other sites exhibiting no such deficiency or much less than the control group. However, we do not feel convinced that the angular stomatitis and the oral fissuring could not have been due to the tobacco, betel and nut chewing habit in the buccal mucosal and anterior tongue cancers.

The gastric juice analysis could be conducted only in our oral and lingual carcinomas. Our pharyngeal and oesophageal cancers had great difficulty in swallowing the Ryle's tube and we were not certain of the reliability of the aspirated material. The difference in the incidence of achlorhydria and hypochlorhydria between the study group and the control group was not striking or constant enough to be of statistical significance in either sex.

We had only one case with a distinct past history of Plummer-Vinson syndrome in our series, and that was in a female who had a carcinoma of the oesophagus at the retro-aortic level. None of our other cases, male or female, gave any history or present evidence of the syndrome.

In the series of over 6000 patients, with malignancy or other disease, that we have so far seen at the Institute only two men and four women presented with the features of Plummer-Vinson syndrome. They had all the classical features of the condition, laryngoscopy and oesophagoscopy excluded any cancer in the pharynx and oesophagus. They are being followed now for periods of one to three years. No cancer has developed, but they are under treatment for their deficiencies and it is hoped cancer will not develop.

The incidence of sideropoenia in the cancer group and in a random clinically healthy population was studied with special care because of the emphasis that Swedish scientists have placed on it.

Sideropoenia did not seem of much aetiological significance in the male though it had a 10 per cent higher incidence in the hypopharyngeal and oesophageal carcinomas than in the controls. For other sites the incidence of sideropoenia was less than in the controls.

In females, sideropoenia was significant in hypopharyngeal carcinomas, its incidence being 100 per cent against a control incidence of 60·27 per cent. The incidence of sideropoenia was not statistically significant for cancers at other sites.

*Diet and nutrition*

TABLE IX.—*Diet*

Figures are percentages

| | Lip | Buccal mucosa | Anterior tongue | Posterior tongue | Pharynx | Hypo-pharynx and cerv. oesoph. | Oeso-phagus | Control |
|---|---|---|---|---|---|---|---|---|
| Vegetarian | 21 | 17·9 | 17·2 | 48 | 31 | 33·3 | 39 | 20 |
| Non-vegetarian | 79 | 82·1 | 82·8 | 52 | 69 | 66·6 | 61 | 80 |

The role of defective diet and nutrition in the genesis of oral and pharyngeal carcinomas has been much discussed. Paymaster (1956) claims that a deficiency of vitamin B complex may play a contributory part. A deficiency of animal proteins, fresh citrus fruits (vitamin C), vitamin B complex and iron in the diet is believed to be the reason for the relatively high incidence of Plummer-Vinson syndrome and of oral and pharyngeal carcinomas in women in Northern Sweden (Wynder *et al.*, 1957).

In South India 97 per cent of the population belong to the labouring and the lower middle class. Their staple diet is rice, either parboiled or polished and therefore deficient in vitamin B. They have usually one vegetable side dish of limited amount. Their diet consists essentially therefore of carbohydrates.

Their diet is deficient in animal proteins, both for the vegetarian and the non-vegetarian. The vegetarian's source of animal proteins is milk which is usually adulterated with water and expensive and only a small quantity of which the people can afford. The non-vegetarian is only so in name. He usually has 2 or 3 small pieces of mutton or a piece of fish, twice or thrice a week. The fish is often of the dried and salted variety. Dhal, another source of protein, is also eaten only occasionally. Melted butter is an occasional luxury and fresh fruits are virtually unknown except on festive occasions, twice or thrice a year. The average diet of the lower middle and labour classes is seriously and equally deficient in calorific value, in proteins, in vitamins and in minerals. As a matter of fact, the great majority of our population have only one fair meal a day.

If, therefore, dietetic deficiencies were to be an important contributory factor in aetiology, oral and pharyngeal carcinomas should be far more frequent than they now are. In this connection, it is significant that if you eliminate the betel, nut and tobacco chewer cancers in our series, the frequency of oral carcinoma is only 3·8 per cent, a little less than the frequency in the far better fed United States.

The present study seems to indicate that malnutrition by itself is not significant as an aetiological factor in carcinomas of the upper alimentary tract.

Food temperature and the amount of spicing used in cooking were found to be of no significance whatsoever.

*Sex*

TABLE X.—*Sex Distribution*

Figures are percentages

| | Lip | Buccal mucosa | Anterior tongue | Posterior tongue | Pharynx | Hypopharynx and cerv. oesoph. | Oesophagus | Control |
|---|---|---|---|---|---|---|---|---|
| Male | 63·2 | 65·9 | 79·3 | 92·1 | 83·8 | 60 | 63·4 | 50 |
| Female | 38·6 | 34·1 | 20·7 | 7·9 | 16·2 | 40 | 36·6 | 50 (Census figures) |

TABLE XI.—*The Sex Ratios for Each Site*

| | Lip | Buccal mucosa | Anterior tongue | Posterior tongue | Pharynx | Hypopharynx and cerv. oesoph. | Oesophagus | Control |
|---|---|---|---|---|---|---|---|---|
| Male : Female | 1·7 : 1 | 1·8 : 1 | 3·8 : 1 | 12 : 1 | 5 : 1 | 1·5 : 1 | 1·7 : 1 | 1 : 1 |

The sex ratio for lip, buccal mucosa, hypopharynx and oesophagus is approximately the same, ranging from 1·5 to 1·9 : 1 in favour of males. The sex ratio begins to rise in the anterior tongue, reaching a peak in the posterior tongue and falling slightly but persisting in favour of males in the oropharynx.

In women tobacco smoking was virtually non-existent. It was interesting therefore to consider the sex ratio after eliminating the pure smokers in males (Table XII).

TABLE XII.—*Sex Ratios for Each Site After Eliminating Male Pure Smokers*

| | Lip | Buccal mucosa | Anterior tongue | Posterior tongue | Pharynx | Hypopharynx and cerv. oesoph. | Oesophagus |
|---|---|---|---|---|---|---|---|
| Male : Female | 1 : 1 | 1 : 1 | 1·2 : 1 | 3 : 1 | 1·3 : 1 | 1 : 2·4 | 1 : 1·5 |

One finds that eliminating the tobacco smokers in males and thus comparing comparable groups, eliminates the male predominance. The persistence of higher male incidence in the posterior tongue was due to the fact that out of the

12 non-smoking posterior tongue male cancers, 11 were betel nut and tobacco chewers and there were only 4 posterior tongue cancers in women.

It is also interesting to note that in Table XII the ratio swings in favour of females in the hypopharyngeal and oesophageal carcinomas.

This data seems to eliminate hormonal influences in differential sex incidence fairly convincingly.

*Social and economic status*

TABLE XIII.—*Social and Economic Status*

| | Lip % | Buccal mucosa % | Anterior tongue % | Posterior tongue % | Pharynx % | Hypo-pharynx and cerv. oesoph. % | Oeso-phagus % |
|---|---|---|---|---|---|---|---|
| Labour | 73·8 | 63·1 | 46 | 32·7 | 32·8 | 36·8 | 13 |
| Lower middle class | 20·9 | 33·7 | 51·7 | 50 | 60·2 | 46·6 | 68·8 |
| Upper middle class | 5·3 | 3·2 | 2·3 | 17·3 | 7 | 16·6 | 18·2 |

The social class distribution for cancers at individual sites in the two sexes was not significantly different.

The approximate class distribution of the South Indian population is : Lower middle class 55 to 60 per cent, Labour 37 to 43 per cent and Upper middle class 2 to 3 per cent.

A study of Table XIII reveals three facts :

i. The percentage of lip and buccal mucosa cancers in labour is significantly higher than their general population trend allows.

ii. The percentage of cancers at the different sites in the lower middle class is in line with their population trend, though the percentage of lingual and pharyngeal and oesophageal cancers is higher amongst them.

iii. The percentage of posterior tongue, pharynx, hypopharynx and oesophageal cancers in the upper middle class is significantly higher than their population trend permits.

The reason for these trends becomes clear immediately the tobacco habits of the three classes are analysed (Table XIV).

TABLE XIV.

| | Labour % | Lower middle class % | Upper middle class % |
|---|---|---|---|
| Betel, nut and tobacco chewers | 78·3 | 51·4 | 33·3 |
| Heavy smokers | 34·3 | 45 | 66·6 |

The tobacco, betel and nut chewing has already been shown to be the essential carcinogenic factor in the buccal mucosa and lip and tobacco smoking either alone or in conjunction with tobacco, betel and nut chewing, to be the main carcinogenetic factor in the tongue, pharynx and oesophagus. The differential frequency of these habits in the different social classes evidently dictates the site incidence of cancer.

*Influence of environment*

The rural and urban distribution of our cancer groups is shown in Tables XV and XVI.

TABLE XV.—*Rural and Urban Distribution of Cancer Cases—Males*

Figures are percentages

| | Lip | Buccal mucosa | Anterior tongue | Posterior tongue | Pharynx | Hypo-pharynx and cerv. oesoph. | Oeso-phagus | Control |
|---|---|---|---|---|---|---|---|---|
| Rural | 58·3 | 71 | 69·9 | 60·5 | 34·3 | 70·1 | 40·3 | 75·6 |
| Urban | 41·7 | 29 | 30·1 | 39·5 | 65·7 | 29·9 | 59·7 | 24·4 |

TABLE XVI.—*Rural and Urban Distribution of Cancer Cases—Females*

Figures are percentages

| | Lip | Buccal mucosa | Anterior tongue | Posterior tongue | Pharynx | Hypo-pharynx and cerv. oesoph. | Oeso-phagus | Control |
|---|---|---|---|---|---|---|---|---|
| Rural | 85·7 | 76·3 | 78 | 25 | 61 | 41·6 | 25 | 75·6 |
| Urban | 14·3 | 23·7 | 22 | 75 | 39 | 58·4 | 75 | 24·4 |

The rural or urban environment seems of little significance in aetiology either in the male or the female. The majority of the sites follow the general population distribution. Any departures from the general population distribution appeared to be accidental ; for example, there were only 4 women with carcinomas of the posterior third of tongue and three of them happened to be urban, thus giving a misleading picture. The only cancer site where there was a consistently higher percentage of urban patients, both in the males and females, and which seemed of significance was the oesophagus. These figures also confirmed a study carried out two years ago. The reason for this is not entirely clear and we do not feel that a higher percentage of heavy smokers in the urban population (77·1 per cent urban smokers against 64·7 per cent rural smokers) in the male could entirely explain this away.

Other factors studied were the frequency of these cancers in the different major religious groups in the country, the influence of heredity, any correlation between blood groups and cancers at the different sites in the upper alimentary tract and the possible role of occupation in aetiology.

*Religion*

TABLE XVII.—*Religion and Cancer Incidence*

Figures are percentages

| | Lip | Buccal mucosa | Anterior tongue | Posterior tongue | Pharynx | Hypo-pharynx and cerv. oesoph. | Oeso-phagus | Control |
|---|---|---|---|---|---|---|---|---|
| Hindu | 98·4 | 90·8 | 80·4 | 80·7 | 87·8 | 80 | 88·2 | 87·24 |
| Moslem | 5·2 | 5·8 | 14·9 | 13·2 | 9 | 16·6 | 9·6 | 9·9 |
| Christian | — | 3·4 | 4·7 | 6·1 | 3·2 | 3·4 | 3·2 | 2·5 |

Religious groupings were of little importance in aetiology.

### *Family history*

A family history of cancer was elicited in the study group for the different sites in the two sexes in 0 to 5·2 per cent of cases whereas the controls gave a family history of cancer in 5·7 per cent of cases. Family history was, therefore, of no significance in aetiology.

### *Occupation*

TABLE XVIII.—*Occupations of Cancer Patients Studied*

| | | % | | | % |
|---|---|---|---|---|---|
| *Males* | Farmers | 32·1 | *Females* | Housewives | 96 |
| | Clerks | 9·7 | | Teachers | 1 |
| | Businessmen | 12·1 | | Unskilled labour | 3 |
| | Teachers | 2·1 | | | |
| | Priests | 1·4 | | | |
| | Unskilled labour (Rickshaw pullers, porters, sweepers and job coolies.) | 37·5 | | | |
| | Skilled labour (Artisans, masons, carpenters, weavers, chauffeurs etc.) | 5·1 | | | |

There was one steam engine driver, one railway guard and one goldsmith amongst the males.

On the whole occupation was of no aetiological significance.

### *Blood group*

An analysis of blood groups showed no significant variation between the study and control group and was not of any aetiological significance.

### *Miscellaneous factors*

A history of chronic pharyngitis or frequent colds was given by only one of our pharyngeal cases. This was therefore not considered of any significance. There was no significant history of trauma or of voice strain.

### CONCLUSIONS

The entire study seems to point to one inescapable conclusion, namely, that betel, nut and tobacco chewing is the factor that is responsible for the high frequency of oral cavity carcinoma in the South Indian population. It is the dominating aetiological factor in cancers of the buccal mucosa, lip and anterior two-thirds of the tongue in both sexes. If the tobacco chewer is excluded, the frequency of oral carcinomas in our series falls to 3·8 per cent, a figure comparable to the frequency in other countries.

Tobacco smoking does not appear to be of much significance in cancers of the buccal mucosa and lip, though there appears to be some contribution. This contribution is reflected in the higher differential sex ratio in the male for cancer at these two sites. But it appears to be of definite aetiological significance in cancer of the tongue, pharynx and hypopharynx (including cervical oesophagus). This is reflected both in the male smoking figures in Table IV and in the sharp rise in the differential sex ratio in favour of males for cancers at these sites.

The overwhelming aetiological significance of the tobacco habit in upper alimentary tract cancers is emphasised in Fig. 3. Heavy habitual users of tobacco in one form or another accounted for virtually the entire male incidence of upper alimentary tract cancers in our series.

Though tobacco dominates the scene, the study reveals that it is *not* the only aetiological agent. This is obvious when the female group is considered. Fig. 3 shows that though the tobacco habit was overwhelmingly important in cancers of the lip, buccal mucosa and tongue in women, nevertheless about 10 per cent of oral cavity cancers, 40 per cent of oropharyngeal cancers, nearly 50 per cent of oesophageal cancers and 83 per cent of hypopharyngeal cancers in women never used tobacco in any form in their lives. Special efforts were therefore made to elucidate this problem. There seemed little doubt that the lower female incidence of cancers in the upper alimentary tract was mainly due to the fewer habitual users of tobacco among them. Hormonal influences did not seem to play a part.

Histories of menstrual irregularities were elicited in detail, clinical evidence of any endocrine disturbance was carefully looked for, and blood chemistry analyses and 17-keto-steroid excretion in urine were studied. Their marital and child-bearing histories were also studied. All the findings were within normal limits and the percentage of variations was similar to that in the controls.

A serum iron level below 90 $\mu$g./100 ml. in males, and 70 $\mu$g./100 ml. in females was considered as sideropoenia. With these standards sideropoenia occurred in about 50 per cent of male hypopharyngeal and oesophageal cancers against a control figure of 37·5 per cent, and in 100 per cent of female hypopharyngeal cancers against a control figure of 60·27 per cent. Whether the 10 per cent difference in males is significant enough to warrant any definite conclusion is questionable. In the female study group the statistical difference is significant but the small number of cancer cases in the group should also be borne in mind. It is because of all these reservations that we hesitate to commit ourselves to an opinion with regard to sideropoenia. However, we are also conscious all the time of the weight of Swedish statistics in favour of the aetiological significance of sideropoenia for cancer at these sites.

There is only one other factor that we consider of some significance in aetiology and that is dental sepsis. It is our impression that though dental sepsis by itself is not significant it does act as a co-carcinogen and assists more powerful carcinogens in the induction of oral cancer.

As we have already stated, avitaminosis is not an aetiological factor though adequate vitaminisation of the individual may exert a protective effect on epithelium.

Inherited influences seemed of little significance in our series. Though genetic influences cannot be totally excluded in carcinogenesis and may explain why one individual with a tobacco habit develops cancer and another does not, the weight of our evidence was against its playing any significant role.

Indians are multiracial and the occurrence of a high rate of oral cancer in the different ethnic groups in India is more suggestive of the influence of habits and environment than of any racial predisposition.

Occupation, economic class, religious groupings, environment, etc., were of no aetiological significance in our series.

We would like to emphasise finally, that statistics, though of vital importance in the elucidation of major factors in causation, can never account for the many casual contributory factors, e.g. there was an undoubted causal relationship between one of our anterior tongue cancers and a ragged tooth. It is possible that syphilis or heavy alcoholism or exposure to irritating fumes may be contributory aetiological factors in an occasional case.

On the whole one salient fact stands out clearly from this study—that the critical factors in carcinogenesis of the upper alimentary tract are " extrinsic " and to that extent carcinomas of these situations fall in the category of " preventable cancers ".

### SUMMARY

Carcinomas of the upper alimentary tract have a very high incidence in South India, with a frequency of a little over 40 per cent of all malignancies seen, whereas the frequencies in Western countries range approximately between 5 to 10 per cent. The reasons for this high frequency were investigated. A total of 1282 individuals comprising 882 cancer patients and 400 non-tumorous and relatively healthy controls were studied. Of the patients 628 were males and 254 females, and of the controls 300 were males and 100 females.

The sites studied were lip, buccal mucosa, tongue, oropharynx, hypopharynx and the oesophagus. The factors analysed were age, sex, religion, occupation, family history, environment, social status, diet, blood groups, habits, pre-existing illness and pre-existing pathology.

The study proved that betel, nut and tobacco chewing was probably the dominating aetiological factor in cancers of the buccal mucosa, lip and anterior tongue in both sexes. The exclusion of the tobacco chewer lowered the frequency of oral carcinomas to 3·8 per cent, a figure comparable to the frequency in Western countries. While tobacco smoking was only of limited aetiological significance in mouth cancers, it assumed a progressively increasing importance as we passed backwards from the lip to the hypopharynx.

The heavy tobacco habit in some form or other accounted for almost all cases of cancer of the upper alimentary tract in males.

In the female, which is a non-smoking group, the tobacco habit was overwhelmingly important in cancers of the oral cavity only. In the pharynx and the oesophagus, the tobacco habit was of only limited significance. The fact that the females were a non-smoking group accounts for the lower incidence of cancer of the upper alimentary tract in them.

Hormonal patterns were of no significance in aetiology, nor did heredity, race, environment, occupation, pre-existing diseases like syphilis, diabetes, hypertension or cirrhosis of the liver or pre-existing pathology like avitaminosis seem of any importance.

However, the contributary significance of dental sepsis and sideropoenia in aetiology was highly suggestive. Sideropoenia seemed especially of importance in hypopharyngeal and oesophageal cancers in the female.

There was only one case of Plummer-Vinson syndrome in our series and one case of a ragged tooth bearing a casual relationship to lingual cancer. Alcoholism was of no significance.

Our thanks are due to Mr. Udayachander of the Department of Biochemistry for the various biochemical analyses, to our ward medical officers for the careful histories elicited and to our records clerks for help in the tabulation of data.

### REFERENCES

ACKERMAN, L. V. and DEL REGATO, J. A.—(1954) *Cancer : Diagnosis, treatment and prognosis*, 2nd edition. St. Louis, Mo., U.S.A. (C. V. Mosby & Co.).
ALSOS, T.—(1960) *Cancer*, **13**, 925.
NIELSEN, J.—(1951) *Ugeskr. Laeg.* **113**, 1742.—(1951) *Ibid.*, **113**, 1752.
PAYMASTER, J. C.—(1956) *Cancer*, **9**, 431.
SANGHVI, L. D., RAO, K. C. M. and KHANOLKAR, V. R.—(1955) *Brit. med. J.*, i, 1111.
SHANTA, V. AND KRISHNAMURTHI, S.—(1959) *Brit. J. Cancer*, **13**, 381.
WYNDER, E. L., HULTBERG, S., JACOBSON, F. and BROSS, I. J.—(1957) *Cancer*, **10**, 430.